# The Efficacy of Tocilizumab in the Treatment of Patients with Refractory Immune-Mediated Necrotizing Myopathies: An Open-Label Pilot Study

**DOI:** 10.3389/fphar.2021.635654

**Published:** 2021-03-16

**Authors:** Sizhao Li, Wenli Li, Wei Jiang, Linrong He, Qinglin Peng, Guochun Wang, Xin Lu

**Affiliations:** Department of Rheumatology, China-Japan Friendship Hospital, Beijing, China

**Keywords:** immune-mediated necrotizing myopathy, tocilizumab, treatment, biomarker, interleukin-6

## Abstract

**Objective:** To evaluate the efficacy of tocilizumab (TCZ) in adult patients with refractory immune-mediated necrotizing myopathies (IMNMs) and investigate possible predictive biomarkers of the response to treatment with TCZ.

**Methods:** Patients with refractory IMNM were enrolled in this open-label pilot observational study and received intravenous TCZ treatment. The clinical response was assessed after 6 months of TCZ treatment according to the 2016 American College of Rheumatology–European League Against Rheumatism (ACR–EULAR) response criteria for adult dermatomyositis and polymyositis. Muscle biopsies were performed to investigate muscle fiber regeneration by immunohistochemical staining of CD56. Serum levels of interleukin (IL)-6 were measured using a multiplex bead-based flow fluorescent immunoassay. The levels of muscle IL-6 mRNA were detected by real-time polymerase chain reaction.

**Results:** A total of 11 patients with refractory IMNM were enrolled in the study, including 3 anti-3-hydroxy-3-methylglutaryl-CoA reductase- and 8 anti-signal recognition particle-positive patients. Seven (63.6%) of these patients achieved clinically significant responses according to the 2016 ACR–EULAR myositis response criteria. Responders had higher baseline serum IL-6 and muscle IL-6 mRNA levels and percentage of CD56-positive muscle fibers than non-responders. Baseline serum IL-6 levels and the percentage of CD56-positive muscle fibers were positively correlated with total improvement score after 6 months of TCZ treatment. Furthermore, muscle fiber necrosis and muscle fiber size variation decreased in repeated muscle biopsies in five responders.

**Conclusion:** Patients with refractory IMNM may respond to TCZ. Baseline serum IL-6 and muscle IL-6 mRNA levels and the percentage of CD56-positive muscle fibers may predict the response to TCZ treatment in these patients.

## Introduction

Immune-mediated necrotizing myopathies (IMNMs) are a novel subgroup of idiopathic inflammatory myopathies (IIMs) characterized by significantly elevated serum creatine kinase (CK) levels, severe proximal muscle weakness, and resistance to conventional therapy (Dalakas, 2015; Allenbach et al., 2018b). Anti-3-hydroxy-3-methylglutaryl-CoA reductase (HMGCR) and anti-signal recognition particle (SRP) antibodies have been recognized as serum biomarkers for IMNM (Allenbach et al., 2018b). Despite intense immunosuppressant treatment, the outcomes of IMNM are worse than those of other IIM subtypes, such as dermatomyositis (DM) and polymyositis (PM). Recent studies have indicated that approximately half of anti-HMGCR- and anti-SRP-positive patients with IMNM still have marked muscle weakness 2 years after aggressive treatment (Pinal-Fernandez et al., 2017; Tiniakou et al., 2017). Therefore, there is a need to discover novel and targeted therapeutics, such as biologics, to improve the prognosis of these patients and investigate the potential targeting mechanism involved in the pathogenesis of the disease.

Interleukin (IL)-6, a pleiotropic cytokine that regulates muscle function, is produced and released locally by skeletal muscle fiber in different pathological conditions. Under physiological conditions, IL-6 maintains muscle homeostasis and positively regulates muscle function (Baeza-Raja and Muñoz-Cánoves, 2004; Serrano et al., 2008). In contrast, it negatively regulates the muscle phenotype under some pathological stimuli (Munoz-Canoves et al., 2013). Pelosi et al. have reported that the treatment of C2C12 myoblasts with recombinant IL-6 significantly inhibits the differentiation of C2C12 myogenic cells (Pelosi et al., 2014). Interestingly, the inhibition of IL-6 activity by an anti-IL-6 receptor (IL-6R) antibody promotes muscle fiber regeneration in both cardiotoxin-induced muscle injury and dystrophin/utrophin double-knockout mice (Fujita et al., 2014; Wada et al., 2017). Allenbach et al. recently demonstrated that upregulated IL-6 expression and impaired muscle regeneration are detected in the muscles of patients with anti-HMGCR and anti-SRP myopathies, which suggests that the IL-6 pathway may be involved in the pathogenesis of IMNM (Allenbach et al., 2018a).

In this pilot study, we evaluated the efficacy of tocilizumab (TCZ), a recombinant anti-IL-6R monoclonal antibody, on both clinical and histological parameters in patients with refractory IMNM and investigate the potential role of IL-6 in the pathogenesis of IMNM.

## Methods

### Patient Selection

Patients with refractory IMNM were enrolled in this study. The inclusion criteria were as follows: 1) anti-SRP- or anti-HMGCR-positive and 2) disease worsening after treatment with high-dose glucocorticoids (equivalent of prednisone 1.0 mg/kg/d for at least 1 month) and at least one immunosuppressant, including azathioprine (AZA), methotrexate (MTX), cyclosporine (CSA), tacrolimus (TAC), or intravenous immunoglobulin (IVIG) at a known effective dose for at least 3 months. Disease worsening was defined as 1) manual muscle testing (MMT) decreasing by ≥ 20% and worsening of physician global activity by ≥ 2 cm on a 10 cm visual analog scale (VAS), 2) worsening of global extramuscular activity by ≥ 2 cm on a 10 cm VAS, or 3) worsening of any three of six International Myositis Assessment and Clinical Studies (IMACS) core set measures (CSMs) by ≥30% (Tjarnlund et al., 2018).

The exclusion criteria were cancer-associated myositis, other connective tissue disease overlap myositis, infection-, drug-, or toxin-induced myopathies, and muscle histopathological findings suggestive of muscular dystrophy, metabolic myopathy, or congenital myopathy.

### Study Design

This study was a 6-month one-arm open-label pilot study. The patients were administered 8 mg/kg intravenous TCZ infused every 4 weeks for six rounds, following a standard dosing protocol. The dose of prednisolone was reduced following a standardized reduction schedule. Stable doses of other immunosuppressive or immunomodulatory agents, including AZA, MTX, CSA, TAC, and IVIG, were administered after TCZ administration.

### Outcome Assessment and Response Criteria

The 2016 American College of Rheumatology–European League Against Rheumatism IIM response criteria for adult DM/PM were used to evaluate the outcomes of patients (Aggarwal et al., 2017). Total improvement score (TIS), which is the sum of the improvement scores in each of the IMACS CSMs and provides a quantitative assessment of improvement for each patient, were calculated 3 and 6 months after TCZ treatment. A TIS of ≥20, 40 and 60 represents minimal, moderate, or major improvement, respectively. A response was defined as a TIS ≥20 at 3 or 6 months.

Overall safety and tolerability of TCZ during the entire treatment period were assessed by adverse events (AEs) and severe AEs (SAEs).

### Detection of anti-HMGCR and anti-SRP Antibodies and Serum IL-6 Levels

The levels of anti-HMGCR antibodies were measured using an enzyme-linked immunosorbent assay (Raybiotech, China) according to a previously described method (Ge et al., 2015). Levels of antibodies against SRP were determined using immunoblotting (EUROLINE, Lubeck, Germany) in accordance with the standard methods (Euroline Myositis Profile 3 immuno line-blot; Euroimmun). The levels of IL-6 in patients’ serum were measured using the multiplex bead-based flow fluorescent immunoassay (Raisecare, Qingdao, China) according to the manufacturer’s instructions.

### Muscle Biopsies and Immunohistochemical Analysis

Muscle biopsies were performed at the baseline and after 6 months. The specimens were frozen in isopentane prechilled with liquid nitrogen and stored at -80°C until processing. Hematoxylin and eosin staining, combined eosin and dystrophin immunostaining (NCL-DYS1, Leica, United Kingdom), and immunohistochemical staining of NCAM/CD56 (ab6123, Abcam, United Kingdom) were performed on 8-μm frozen sections as previously described (Arouche-Delaperche et al., 2017; Allenbach et al., 2018a). The half area of each section was quantitative analyzed. For each patient, the total number of muscle fibers and necrotic and regenerating muscle fibers was manually determined. The Feret diameter of each muscle fiber outlined by the immunostaining of dystrophin was automatically measured using ImageJ v1.51u. Myofiber necrosis, defined as pale and/or hyalinized muscle fibers combined with the loss of sarcolemmal integrity/coarse appearance, was evaluated on combined eosin and dystrophin immunostaining (Aggarwal et al., 2017). NCAM/CD56-positive fibers represent regenerating muscle fibers.

### RNA Extraction and Quantitative Real Time-Polymerase Chain Reaction (qPCR)

Total muscle RNA was extracted using TRIzol reagent (Thermo, Waltham, United States). RNA was reverse-transcribed into cDNA using a PrimeScript™ RT reagent kit with gDNA Eraser (Takara, Dalian, China). qPCR was performed using the TB Green Fast qPCR mix in a 7,500 Real-Time PCR system (Applied Biosystems, Singapore) according to the manufacturer’s instructions. The expression levels of IL-6 were normalized to those of GAPDH.

The primers used were as follows: IL-6: forward 5′-GAA​AGC​AGC​AAA​GAG​GCA​CT-3′ and reverse 5′-AGC​TCT​GGC​TTG​TTC​CTC​AC-3′; GAPDH: forward 5′-CCT​CCT​GCA​CCA​CCA​ACT​GCT​T-3′ and reverse 5′-GAG​GGG​CCA​TCC​ACA​GTC​TTC​T-3′.

### Statistical Analysis

Statistical analysis was performed using SPSS 22.0. Differences between responders and non-responders were compared using the Mann-Whitney U test. To compare the parameters before and after TCZ treatment, a paired-samples *t*-test was used. The correlation between clinical outcome and biomarkers was analyzed using Spearman’s rank correlation. *p* < 0.05 was considered significant.

## Results

### Baseline Characteristics of Patients

A total of 11 patients were enrolled in this study. The mean age of disease onset was 42 years (range, 34–81 years) and seven females and four males were included. Eight were anti-SRP-positive and three were anti-HMGCR-positive. All patients had muscle weakness and significant CK level elevation. Among these patients, 54.5% had complications with dysphagia, 18.2% had interstitial lung disease, and 9.1% had a skin rash. All patients were initially treated with high-dose glucocorticoids and/or immunosuppressants for more than 3 months before enrollment (Table 1).

**TABLE 1:** Baseline demographics and clinical characteristics of patients with IMNM.

Variables^a^	All patients (n = 11)
Sex ratio, (F/M)	7/4
Anti-SRP positive, n (%)	8 (72.7%)
Anti-HMGCR positive, n (%)	3 (27.3%)
Age at symptom onset, years	42 (34–53)
Muscle weakness	11 (100%)
Max CK before any treatment, IU/L	7,411 (4,375–10,891)
Dysphagia, n (%)	6 (54.5%)
Interstitial lung disease, n (%)	2 (18.2%)
Skin rash, n (%)	1 (9.1%)
Duration of previous therapy, months	6 (5–8)
Previous medication	
Initial treatment of high-dose glucocorticoid, n (%)	11 (100%)
Methotrexate, n (%)	6 (54.5%)
Azathioprine, n (%)	3 (27.3%)
Cyclosporine, n (%)	1 (9.1%)
Methotrexate and tacrolimus, n (%)	1 (9.1%)
Additional IVIG besides immunosuppressant, n (%)	4 (36.4%)

^a^ Presented as median (interquartile range).

IMNM, immune-mediated necrotizing myopathy; SRP, signal recognition particle; HMGCR, 3-hydroxy-3-methylglutaryl-CoA reductase; CK, creatine kinase (normal range: 26–200 IU/L); IVIG, intravenous immunoglobulin.

### Clinical Response to Tocilizumab Treatment

Seven (63.6%) of these patients achieved the threshold of a minimal clinically significant improvement after 3 months of treatment with TCZ and were classified as responders. Four (36.4%) were non-responders and none worsened 6 months after TCZ treatment. Four (36.4%) and three (27.3%) patients attained a moderate and major improvement after 3 months of treatment with TCZ, respectively. At 6 months, seven (63.6%) patients achieved a major improvement. The median TIS was 50 and 75 at 3 and 6 months in all patients, respectively. All six IMACS CSMs except extramuscular global activity improved at 3 and 6 months compared with the baseline for the whole patient population (Table 2).

**TABLE 2:** Six IMACS core set measures in patients with IMNM at the baseline and after 3 and 6 months of treatment with tocilizumab.

Variable^a^	Baseline	Month 3	Month 6
Physician global activity, VAS (10 cm)	6.0 (5.7–6.5)	5.0 (3.8–5.5)	3.0 (1.5–5.5)
Patient global activity, VAS (10 cm)	7.0 (6.0–7.0)	4.2 (4.0–6.0)	2.5 (2.3–6.0)
MMT-8 (0–80)	49 (42–52)	51 (49–58)	60 (53–65)
HAQ (0–3)	1.6 (1.1–1.85)	0.9 (0.6–1.3)	0.55 (0.3–0.85)
CK, IU/L (26–200)	975 (730–1751)	491 (185–702)	240 (86–416)
Extramuscular activity, VAS (10 cm)	2.0 (1.5–2.2)	2.0 (1.5–2.2)	2.0 (1.5–2.2)

^a^ Presented as median (interquartile range).

IMNM, immune-mediated necrotizing myopathy; IMACS, International Myositis Assessment and Clinical Studies; VAS, visual analog scale; MMT-8, Manual Muscle Test-8; HAQ, Health Assessment Questionnaire; CK, creatine kinase (reference: 26–200 IU/L).

### Biomarkers for Predicting the Outcomes

**FIGURE 1:**
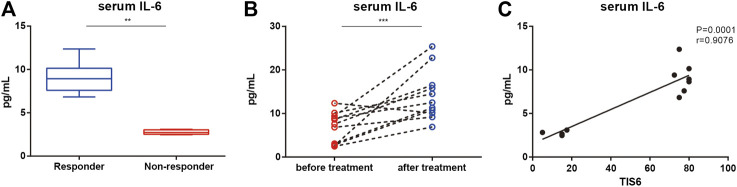
Serum levels of interleukin (IL)-6 and its association with the clinical response of patients with immune-mediated necrotizing myopathy before and after treatment with tocilizumab (TCZ). **(A)** Serum levels of IL-6 between responders and non-responders at the baseline. **(B)** Serum levels of IL-6 of patents before and after 6 months of treatment with TCZ. **(C)** Correlation of serum IL-6 levels of patients at the baseline and the total improvement scores (TIS) after 6 months.

The serum IL-6 levels of responders at the baseline were higher than those of non-responders (*p* = 0.008; Figure 1A). In all patients, serum IL-6 levels were higher after 6 months of treatment than at the baseline (median 14.5 pg/ml, range 9.2–25.4; Figure 1B) but there was no difference between responders and non-responders. There was a positive correlation between the baseline serum IL-6 level and TIS after 6 months (Figure 1C).

**FIGURE 2:**
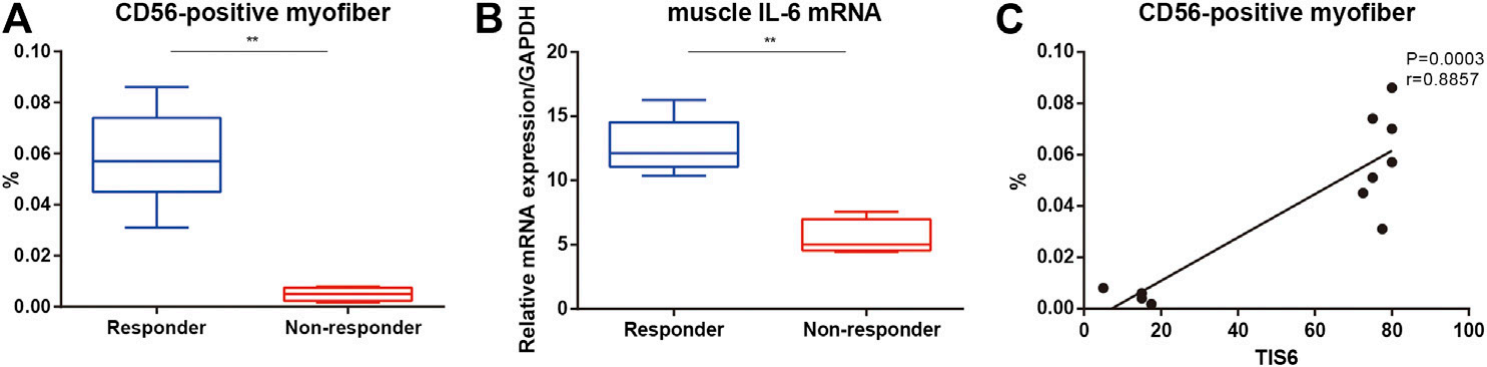
CD56-positive muscle fibers and muscle interleukin (IL)-6 mRNA levels in patients with immune-mediated necrotizing myopathy. **(A)** Percentage of CD56-positive muscle fibers between responders and non-responders at the baseline. **(B)** Muscle IL-6 mRNA levels between responders and non-responders at the baseline. **(C)** Correlation of the percentage of CD56-positive myofibers at the baseline and the total improvement scores (TIS) after 6 months.

Responders had a higher baseline muscle IL-6 mRNA level and percentage of CD56-positive muscle fibers than non-responders (Figures 2A,B). There was a positive correlation between TIS after 6 months of TCZ treatment and the baseline percentage of CD56 positive fibers in all patients (Figure 2B).

**FIGURE 3:**
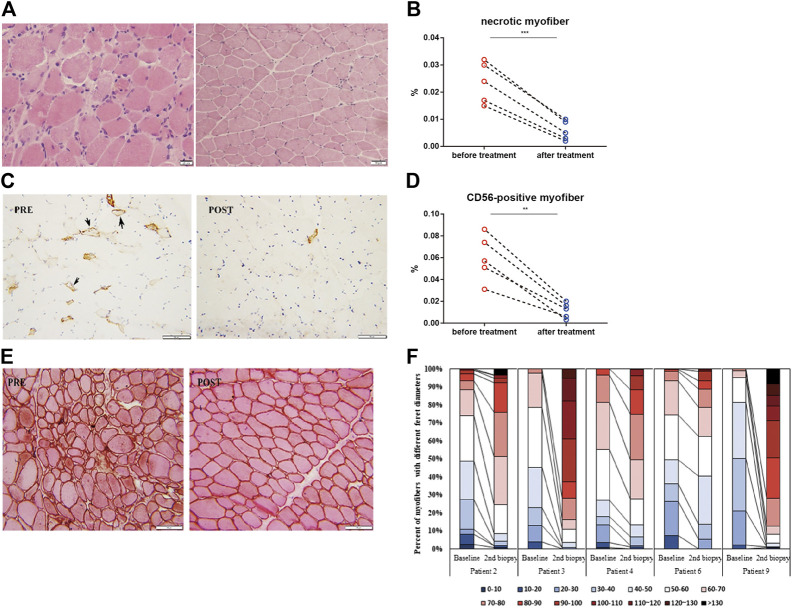
Histopathological changes in muscle biopsies in the responder group (n = 5; 4 anti-SRP-positive, 1 anti-HMGCR-positive) before and after 6 months of treatment with tocilizumab. **(A** **,** **C)** Hematoxylin and eosin staining **(A)** and immunohistochemical staining of CD56 **(brown)** **(C)** in muscle biopsy of one anti-SRP-positive patient before (PRE) and after (POST) treatment with tocilizumab. **(B,D)** Changes in the percentage of necrotic myofibers **(B)** and CD56-positive muscle fibers **(D)** before and after treatment with tocilizumab among five responders. **(E)** Combined eosin and dystrophin immunohistochemical staining in muscle biopsy of one anti-SRP-positive patient before (PRE) and after (POST) treatment with tocilizumab. **(F)** Quantification of the percentage of muscle fibers with different Feret diameter and their distribution before and after treatment with tocilizumab (Scale bar, 20 µm).

Repeated muscle biopsies were performed in five patients who responded to treatment with TCZ. After 6 months of treatment, the percentage of necrotic muscle fibers decreased from 2.36 ± 0.76 to 0.6 ± 0.39% (*p* = 0.0028; Figures 3A,B). The percentage of regenerating muscle fibers in post-treatment muscle biopsies was significantly lower than that in pre-treatment muscles (5.98 ± 2.12% vs. 1.16 ± 0.7%, *p* = 0.0007; Figures 3C,D). The percentage of myofibers with a Feret diameter of less than 40 µm significantly decreased after treatment with TCZ, leading to a redistribution of muscle fibers with a Feret diameters between 40 and 100 µm (Figures 3E,F).

### Safety Data

There were 10 transient AEs in five patients, namely, mild hypofibrinogenemia without bleeding (n = 2), antifungal cream-treated tinea corporis (n = 1), oral antibiotic-treated respiratory tract infection (n = 2), allergic rash (n = 2), and leukocytopenia (n = 3). Only hypofibrinogenemia was considered to be related to TCZ. All these AEs were mild and none resulted in TCZ dose reduction or withdrawal. There were no SAEs.

## Discussion

In this preliminary study, we demonstrated that 7 out of 11 patients with refractory IMNM responded well to treatment with TCZ, and 63.6% achieved a major improvement according to the new response criteria for myositis improvement after 6 months of TCZ treatment. Furthermore, baseline serum IL-6 levels, muscle IL-6 mRNA levels, and the percentage of regenerating muscle fibers (CD56-positive myofibers) may be effective markers to predict the response to TCZ treatment. Additionally, muscle biopsies indicated that the redistribution of muscle fibers with a Feret diameter between 40 and 100 µm after TCZ treatment suggest a positive effect of TCZ treatment on the regeneration of muscle fibers.

TCZ has been widely used in the treatment of various refractory autoimmune disorders, such as giant cell arteritis and rheumatoid arthritis (Smolen et al., 2008; Stone et al., 2017). Three previous case reports have suggested that IL-6 blockade is effective in IIM (Narazaki et al., 2011; Kondo et al., 2014; Beaumel et al., 2016). In this prospective study, we demonstrated that an IL-6R antagonist was effective in the clinical improvement of patients with refractory IMNM. In the muscle biopsy, we compared baseline IL-6 mRNA levels between responders and non-responders. Interestingly, the baseline muscle IL-6 mRNA levels in responders were higher than those in non-responders, which suggests that muscle IL-6 mRNA may be a useful marker for the prediction of therapeutic response. However, there was no correlation between the change in muscle IL-6 mRNA levels before and after TCZ treatment, which may be related to the small number of muscle biopsies after treatment. In the future, we will further investigate the relationship between muscle IL-6 mRNA and TCZ action in paired and large samples to estimate the role of IL-6 in IMNM.

In this study, we focused on the characteristics of repeated muscle biopsies in five patients who responded well to TCZ treatment. Before TCZ treatment, all patients received aggressive therapy. Although previous immunomodulatory therapy resulted in a significant decrease in CK levels in these patients, the patients continued to clinically deteriorate with muscle weakness and a very low MMT-8 score. Previous studies have established that there is a discrepancy between decreased CK levels and worsened muscle strength in patients with muscular disorders. A common explanation for this phenomenon is that muscle strength improvement could be delayed by muscle fiber regeneration. A previous study demonstrated that muscle remodeling occurs up to 30 days after a single electrical stimulation (Mackey et al., 2011). In these five patients, the average duration of routine immunotherapy before the first muscle biopsy was 5 months, which was sufficient to complete the process of muscle regeneration. However, we still observed the prominent and excessive regeneration of muscle fibers with a significant decrease in muscle fiber diameter. In previous basic research, these pathological features were reported when myoblast fusion was disturbed during muscle regeneration (Hindi et al., 2017). In this study, we detected a higher level of muscle IL-6 mRNA and percentage of regenerating muscle fibers in responders than in non-responders. Moreover, we observed a positive correlation between the percentage of CD56-positive myofibers and clinical improvement score, and muscle fibers with a Feret diameter between 40 and 100 µm were redistributed and regenerated after TCZ treatment. Taken together, these results suggest that long-lasting elevated IL-6 levels may lead to severe muscle weakness via the reduction of myoblast fusion during muscle regeneration. Blocking the IL-6 signal using TCZ could recover muscle regeneration and improve muscle performance in patients with IMNM.

However, there are limitations to this study. First, the open-label design of this study may have affected the objective evaluation of the disease by physicians and patients. Second, owing to the small number of patients included in this study and lack of a control arm, caution is required in the interpretation of the results.

In conclusion, TCZ treatment may be beneficial for patients with refractory IMNM. The response to TCZ can be predicted by baseline serum IL-6 and muscle IL-6 mRNA levels and the percentage of CD56-positive muscle fibers in these patients. Additionally, this study provides new insights into the role of IL-6 in the pathogenesis of IMNM. In the future, a randomized placebo-controlled trial of IL-6 blockade involving more patients with IMNM is needed to confirm our results.

## Data Availability

The original contributions presented in the study are included in the article/Supplementary material, further inquiries can be directed to the corresponding author.
